# Performance evaluation of a hybrid sequencing batch reactor under saline and hyper saline conditions

**DOI:** 10.1186/s13036-019-0192-1

**Published:** 2019-07-29

**Authors:** Mostafa Tizghadam Ghazani, Alireza Taghdisian

**Affiliations:** 1 Faculty of Civil, water and Environmental Engineering, Shahid Beheshti University, Tehran, Iran; 2 Environmental Engineering, Tehran, Iran

**Keywords:** Hybrid sequencing batch reactor, Biological process, Saline wastewater, Hybrid growth, Biofilm, Suspended sludge

## Abstract

Significant rise in concentration of saline wastewater entering the treatment plants has been resulting in many problems in the biological treatment processes. On the other hand, the specific conditions of physicochemical treatment methods for saline and hyper saline wastewater have limited their application on a large-scale. Over the past few decades, Sequencing Batch Reactor (SBR) process has been widely used as an efficient, well-designed and practical approach for treatment of domestic and industrial wastewater due to its cost-effectiveness and simplicity. SBR Performance can enhance by providing simultaneous suspended and attached growth of microorganisms which act as a hybrid growth. In this study, a lab-scale Hybrid Sequencing Batch Reactor (HSBR) with 6.4 l working volume was used to examine the effect of salinity (NaCl), increased from 0 to 6.7% (g NaCl/ L wastewater), on the biological treatment. Therefore, COD, MLSS, MLVSS and SVI parameters have been measured over a period of 7 months of operation. The operational parameters namely pH, dissolved oxygen (DO) and temperature were 7.5–8.5, 1.5–6.8 mg /L and 20–25 °C respectively during whole experiment. Influent COD of synthetic wastewater was maintained at 650 ± 25 mg/L. The HSBR Cycle time including, influent feeding, React, Settling and effluent discharge were 1/20/1/1 h respectively. Results indicated that by increasing salt concentration from 0 to 67.7 g NaCl/L, the COD removal efficiency reduced from 94.22 to 53.69%. Moreover, as the NaCl concentration increased, MLSS rose up to 69%, while MLVSS almost stayed constant and SVI dropped by 83%. The results indicated that the simultaneous use of suspended and attached growth of microorganisms and gradual increasing of salt content of wastewater could lead to greater biomass concentration and ultimately improvement in the degradation of organic matter. Besides, settling performance and its velocity were noticeably improved by increasing salinity.

## Introduction

Global human population growth has been resulting in progressive development of industries (e.g. cheese manufacturing, marine food manufacturing, papermaking, pharmaceuticals process and oil and gas plants). The upshot of using large extent of inorganic salts (mostly NaCl) in various said industries is immense increasing of salinity in their discharge. On the other hand, water scarcity and direct use of seawater in many areas, especially coastal cities, has been leading to discharge of large amount of saline wastewater into the wastewater network (Chen, Y. et al., 2018) [1]. Furthermore, infiltration of subsurface water in coastal area, landfill leaches and contaminated groundwater are other sources for increasing dissolved solids in wastewater (Moussa et al., 2006) [2]. As a result, the inflow of saline and hyper saline wastewater to the treatment plants has been considerably going up, as much as 5% of the global wastewater treatment streams (Lefebvre et al., 2007) [3].

Wastewater classification into saline and hyper saline is based on the amount of total dissolved solids exist in the wastewater stream (Shi et al., 2012) [4]. Since NaCl has the greatest impact on measuring salinity, in most of the previous researches salinity of wastewater was measured based on the amount of NaCl (wt.%) dissolved in wastewater. As a technically feasible classification, hyper saline and saline wastewater contains at least 35 and 10 g of NaCl in one liter of wastewater, respectively (He, H., et al., 2016) [5].

In general, there are two solutions for treatment of saline wastewater: 1) physicochemical methods, such as adsorption technique, membrane system, ion exchange, electro dialysis etc. which have disadvantages like high costs, special application conditions, secondary pollution which needs to be retreated and complex technology; 2) Biological methods that are cost-effective, have a simple and flexible process and also they have shown a high removal efficiency. (Fan et al., 2011 [6]; Neilly et al., 2009 [7]; Dincer and Kargi, 2000 [8]). Biological systems could be categorized into two major proccesses; a) continuous and b) discontinuous. Compared to Continuous system, discontinuous biological treatment operation like Sequence Batch Reactor (SBR) has better removal efficiency and flexibility of the process. Also, the use of one tank for whole process diminishes the adverse footprint of the whole system on the environment. (Tzahi Y. Cath et al., 2016) [9].

Although biological treatment processes have many advantages, but there are some inhibitory factors that could cause poor performance of these approaches. Salinity, mainly NaCl, is one of the most important of these factors. High concentrations of salt in the influent wastewater could cause shock to microorganisms, reduces cellular enzymes activity and ultimately could lead to plasmolysis of the cells (Uygur, 2006; [10] He, H., et al., 2016 [5]). In addition to detrimental effects of salinity on microorganisms, it could result in physical and biological changes in suspended sludge and biofilm, including sedimentation, bio flocculation and Extra Polymeric Substance (EPS) contents (Chen, Y. et al., 2018) [1].

There are numbers of techniques that could possibly minimize the destructive impacts of salinity on the activity of microorganisms, comprised of the use of hybrid growth (attached and suspended), gradual salt introduction to the system for bacterial acclimatization, and addition of cultivated microorganisms like salt-resistance and halophile bacteria into conventional activated sludge process. (Rene et al., [11] 2008; Kulkarni, 2013; [12] Figueroa et al., 2008 [13]). Hybrid Sequencing Batch Reactor (HSBR) is an enhanced system of conventional SBR, in which two types of bacterial growth, suspended and attached growth (biofilm) are occurred simultaneously in a single bioreactor (Mielcarek et al., 2015) [14]. The HSBR system has many advantages over the conventional SBR method, including the ability to grow different types of bacteria, better resistance to inhibitory effects such as salinity, greater biomass retention and lower reactor volumes (Wang et al., 2016 [15]; Yusoff et al., 2016 [16]). Biomass carriers, or media, act as a bedding for attached growth. These carriers have a high level of specific surface area and lower density than wastewater fluid (Arnaiz et al., 2007) [17]. Non-uniform structure of the biofilm and the different amounts of oxygen within its layers allows the various bacteria to grow. For instance, in the deeper layers of the biofilm, anoxic conditions exist, which is desirable for denitrifiers bacteria growth (She et al., 2016) [18]. In such a system the removal efficiency of organic and nutrition matters could be improved due to biofilm simple, flexible and stable structure (Xia et al., 2008) [19].

Although two aforementioned microbial growth occur in a one single reactor, but they act very different in removing of nutrient and organic matters from the wastewater. The population structure and type of bacteria exist in suspended sludge and attached biofilm change in different salinity (Wang et al., 2016) [15].

Studies have shown that the application of attached growth could improve the performance of biological saline wastewater treatment. Wang et al. (2016) [15] studied the effect of salinity on microbial activity and microbial community in a HSBR system. They found that the extracellular polymeric substances (EPS) value in biofilm is higher than suspended sludge. EPS is the most significant structure of biofilm which sticks to the surface of microorganisms and forms a protective shield. As a result, biofilm has more resistance to saline environment than suspended sludge. She et al. (2016) [18] examined the effects of different salinity on nitrogen removal efficiencies in HSBR system and indicated that hybrid system had a 10% more efficiency at 9.8 g NaCl/L than conventional SBR system. Yusoff et al. (2016) [16] evaluated the performance of two SBR systems: one with suspended growth and the other with hybrid growth. They observed 18% better COD removal efficiency of hybrid growth compared to the other system.

As a matter of fact, low amount of salinity may increase organic removal efficiency. Uygur, A. (2006) [10] studied the biological nutrient removal of saline wastewater in SBR and found that the removal efficiency of organic matter in low salinity was improved because of stimulatory effect on bacteria. Shi et al. (2012) [4], also showed that the best performance of salt-resistance bacteria is in salinity of 1%.

Sharp increase of salinity significantly affects the biological performance of bacteria. Uygur, A., Kargi, F., (2004) [20] founded that in the SBR system, when salinity increase from 0 to 6%, the COD removal efficiency dramatically decreased from 96 to 32% respectively. Wang et al. (2016) [15] showed that by increase of salinity from 0 to 8%, COD removal efficiency significantly dropped from 95 to 35%. Chen, Y. et al. (2018) [1] used the conventionasl SBR system to treat saline wastewater and illustrated that organic removal rate decreases from 95 to 56% when salinity rose up from 0 to 2%, respectively.

Thus, the main purposes of the current study are to describe the effects of salinity (0–67 g NaCl/l) on the performance of the HSBR system. Therefore, COD removal rate, MLSS, MLVSS, SVI and settling velocity were measured to ascertain the adverse effect of salinity on the performance and activity of microorganisms, microbial population and settling properties.

## Materials & methods

### Reactor setup

**Fig. 1:**
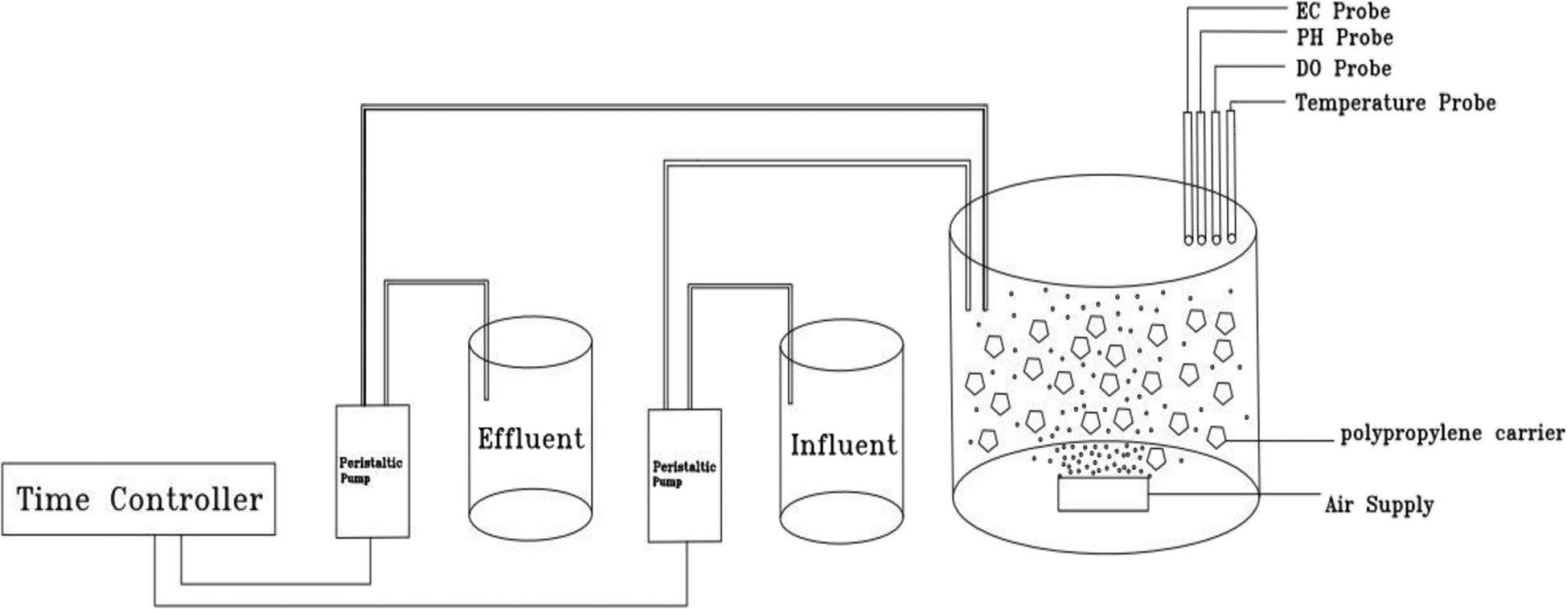
Schematic diagram of HSBR system

A Pilot-scale Plexiglas HSBR system with circular cross section was used for this study (Fig. 1). The HSBR had a working volume of 6.4 L with dimension of 18 cm for diameter and 30 cm for its height. Polyethylene moving carriers with an average specific surface area of 500 m2/m3 and density of 95 kg/m^3^ were used as media for attached growth of biofilm. Two identical peristaltic pumps and three analogue time switches (Theben Germany) were used for controlling influent and effluent wastewater. The HSBR was operated in 24 h working cycle including 1 h influent feeding, 20 h reaction (aeration) time, 1 h settling and 1 h decanting. The activated sludge seeds were obtained from Shahid Beheshti University (SBU) municipal wastewater treatment plant located in north of Tehran (Iran). System was introduced by 3250 mg/L of MLSS as the start-up seed. The dissolved oxygen (DO) concentration, pH and Temperature in the system were maintained between 2.6–6.8 mg/L, 7.2–8 and 18–26 °C respectively throughout the study.

### Synthetic wastewater

This study was carried out with synthetic wastewater which was prepared in daily basis with tap water. The Molasses was used as an only substrate with constant concentration of 750 ± 10 mg/L which produced influent COD of 650 ± 30 mg/L. Additionally, other synthetic wastewater substances including NH_4_Cl, KH_2_PO_4_, KCl, Salt (NaCl) and necessary Trace elements for microorganism growth were added to the synthetic wastewater (She et al., 2016) [18]. The COD/N/P ratios of synthetic wastewater was kept constant at 100/6/2. A specific amount (0–67.7 g/L) of NaCl was added to the synthetic wastewater for providing salinity. Due to negligible effects of other minerals in salinity of wastewater, above-mentioned amount of NaCl was considered as the total salinity of the wastewater. (Zhou et al., 2012) [4].

### Experimental procedure

At the beginning, HSBR was introduced by non-salt acclimated microorganisms from an aeration tank which were obtained from SBU Wastewater Treatment Plant. The initial activated sludge had the following physical Properties of MLSS 3250 mg/ L, MLVSS 2760 mg/L and SVI of 156 mL/g. In every cycle, bioreactor was fed with 3 L of fresh wastewater and at the end of settling period, 3 L of limpid supernatant was pumped out and without delay next nutrient media was fed into the bioreactor for next treatment operation. Sludge age was adjusted to 30 days by the amount of wasted sludge removed from mixed reactor during each cycle and the biomass in the effluent.

For about 2 months, the system worked with zero-salt level in influent feed. Then 5 g/L of NaCl was added to the influent wastewater for about 2 weeks. This step was followed by increasing salinity in a step-by-step order to 10, 20, 30, 40, 50 and finally to 67.7 g/L when steady state conditions were observed. Required time to reach steady state conditions was different in each salt increments. The wastewater and sludge samples were frequently withdrawn from reactor at least 3 times a week in order to analyze the COD, MLSS, MLVSS and SVI.

### Analytical methods

COD, MLSS, MLVSS and SVI parameters were determined as stated by standard methods [21]. A DR1900 Portable Spectrophotometer (HACH, USA) was used for measuring COD values. Excessive amounts of chlorine ion result in a substantial error in determination of COD value. Therefore, in this study a modification of standard methods was used and sufficient amount of mercury sulfate was added to the samples before COD tests [22–24], and. The operational parameters namely DO, pH, Temperature and Electric Conductivity (EC) were measured by a digital multi-meter (SensoDirect 150, Germany).

## Result and discussion

### COD removal efficiency

Figure 1 shows treated effluent COD values of the HSBR during the whole experiment at different levels of salinity. For the purpose of biofilm formation on the suspended carrier and reaching to the steady state conditions, the influent wastewater with zero salt level was fed to the system for about 60 days. At the end of this period a slight improvement in COD removal rate was observed and COD removal rate was reached to 93.58%. Following to the mentioned period, the influent salinity increased by 5 g NaCl/L and was kept constant for about 15 days to reach steady state conditions. At the end of this period, COD removal rate was increased to 94.22% which was the highest removal efficiency observed in this study. The reason for this little improvement was stimulatory effect of salt on activity of microorganisms. This finding that a small amount of salt (below 10 g NaCl/L) is in favor of bacterial growth and reproduction is in line with other studies. Zhang et al. (2010) [25] studied the effect of salt on performance of a SBBR system and showed that the maximal nutrient removal rate happened when salt concentration maintained at 10 g NaCl/L. Chen et al. (2018) [1] investigated the effect of salt on a SBR system and indicated that the influence of salinity on bacteria was bearable at low salinity (< 10 g/L). She et al., (2016) [18] assessed the performance of the SBBR under saline condition and found that increase of salinity (1.4–4.2 g NaCl/L) promoted nitrification and denitrification ability. Amin et al. (2014) [26] also investigated the bacterial adaption to salinity by using an SBR system and showed the maximum removal efficiency of COD was occurred at 4 g/L of NaCl concentration. They pointed that adaptation of biological population to saline environments may result in higher efficiency of biological system.

**Fig. 2:**
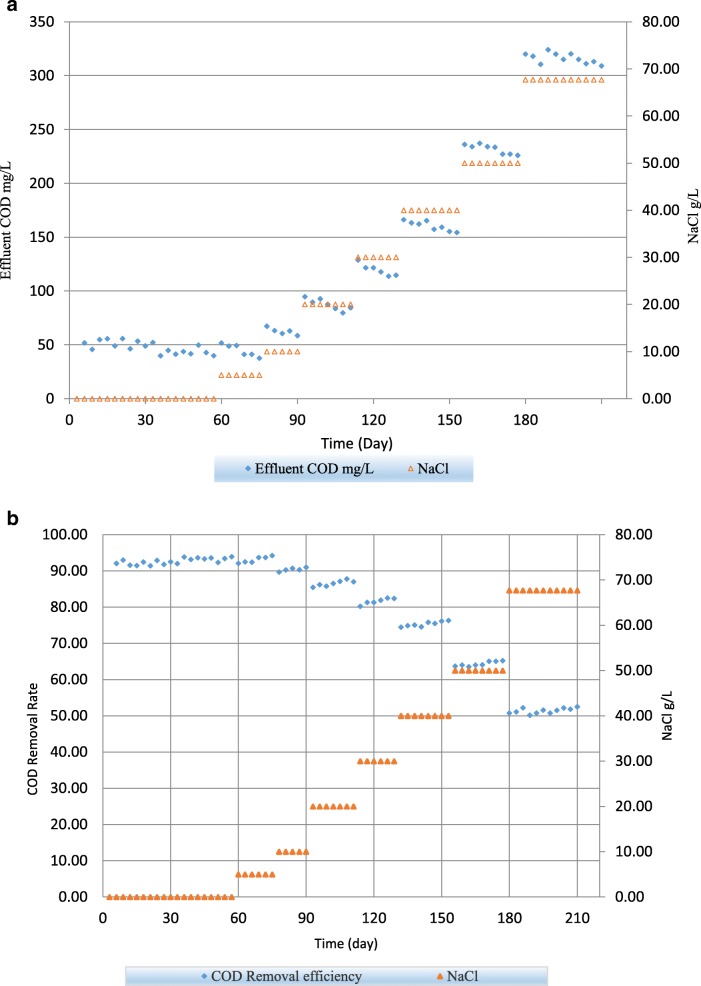
**a** The effluent COD (mg/L) under different salinities. **b** COD removal efficiencies

Following to the previous period, the influent NaCl content was increased to 10 g/L. At the end of this period and after about 12 days to reach steady state conditions, HSBR COD removal efficiency was met a good condition by showing a 91% removal of organic pollutants. To define the steady state conditions in each salinity level, the reactor performance was monitored in terms of COD, MLSS and MLVSS. By increasing NaCl in a stepwise series to 20, 30, 40, 50, 67.7 g/L, the COD removal efficiencies dropped to 87.01, 82.36, 76.27, 65.22 and 52.46% respectively. Figure 2 represent the COD removal rate proportion to each salinity level. The adverse inhibition effect of salinity causes significant decrease in COD removal efficiency. At the last salinity level of 67.7 g NaCl/L, after about 30 days to observe the steady state conditions, the COD removal efficiency dropped at the rate of 41.76% compared to the highest efficiency at 5 g NaCl/L. The high concentration of salt causes loss of cellular activities, dehydration and inhabitation of many active enzymes in biological treatment. However, system showed a good performance for removal of organic matters compared to conventional SBR system even at 30 g NaCl/L. Yusoff et al. (2016) [16] used two identical SBR system, one with attached growth named hybrid granular SBR, and the other was a conventional SBR named suspended granular SBR system. He observed 18% higher efficiency for COD removal in hybrid system. Wang et al. (2016) [15] used SBBR system and stated that by increasing salinity from 0 to 70 g NaCl/L the COD removal rate decreased significantly from 96 to 42% respectively. Chen, Y. et al. (2018) [1] used SBR system for examination of salinity on performance of biological treatment and he observed that COD removal efficiency shifted downward from 95 to 57% proportional to 0 and 20 g NaCl/L respectively.

Uygur, A., Kargi, F., (2004) [20] also, used SBR system and they showed that with increasing salinity from 0 to 6%, removal efficiency of COD dropped significantly from 96 to 32%. The results of this study show that the removal efficiency of organic pollutant was inhibited less severely than those reported at equal levels of salinity. This might be result from the diversity of biological treatment process, variety of microorganism in biomass and influent wastewater. Moreover, it could be attributed to the biofilm special 3D-structure and capabilities for growth and reproduction of many kind of bacteria. Attached growth of microorganisms has a lot of distinct advantages over suspended growth. First, it could stimulate multi-cultural bacterial growth and consequently causes high active biomass concentration (Yusoff et al., 2016) [16]. Second, biofilm unique structure could improve the interaction of substrate with microorganisms by proving a multiple reaction site. Third, as suggested by other articles, some protective substance like organic polymer which secreted by microorganism and acted as a defense mechanism in harsh environment, could grow more easily in biofilm than suspended sludge, and therefore salinity has more significant inhabitation on suspended sludge compared to biofilm. (Wang et al. (2016) [15] She et al., (2016) [18]).

### MLSS and MLVSS variation

**Fig. 3:**
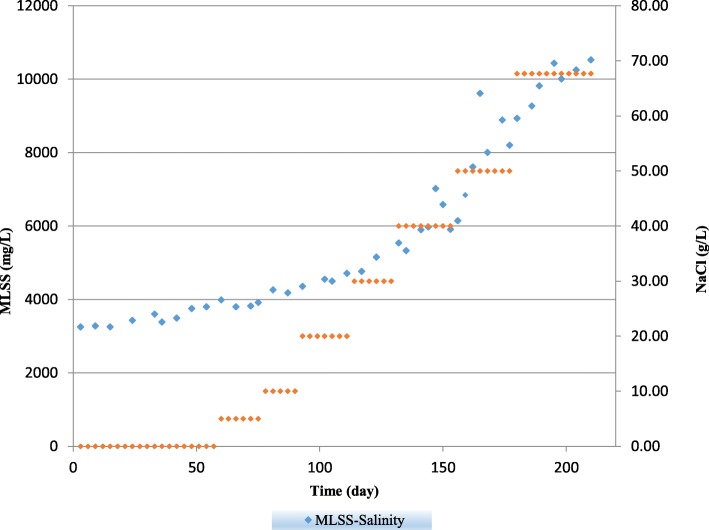
The Variation of MLSS values during whole experiment

In this study, MLSS and MLVSS were measured to represent the mass of microbes that exist in bioreactor. As shown in Fig. 3, the MLSS values were slightly increased with increase of salinity to 20 g NaCl/L. by increasing salinity further to 67.7 g NaCl/L, MLSS reached to its highest level at 10,530 mg/L. it means that MLSS increased about 69% from the beginning of the study.

This result could be attributed to the definition of MLSS. The total weight of biomass comprise of viable microbes, dead cells and other organic matter (Frolund et al., 1996) [27]. By increasing salinity, various kinds of species started to suppressed and as a result, the number of dead cells increased at higher rate compared to non-saline environment. On the other hand, slat-resistant microorganisms like halotolerant and halophilic species, have a chance to grow more favorably. In fact, some species could grow better in biofilm than suspended sludge (Wang et al., 2016) [15]. As a result, the total amount of these specific organic matters increased with increase of salinity. Yusoff et al. (2016) [16] observed 52% increase in MLSS by using hybrid SBR. They explained that the co-existence of biofilm and suspended sludge could promote the bacterial growth and reproduction and consequently causes higher MLSS and MLVSS. In another study by (Alipour et al., 2016) [28] they used a biological aeration batch method with suspended growth for treatment of saline wastewater and they found that suspended solids were increased with increase of salinity and stated that the conversion of dissolved solid to suspended solid as a result of biological deposition in high saline environment could give rise to MLSS.

**Fig. 4:**
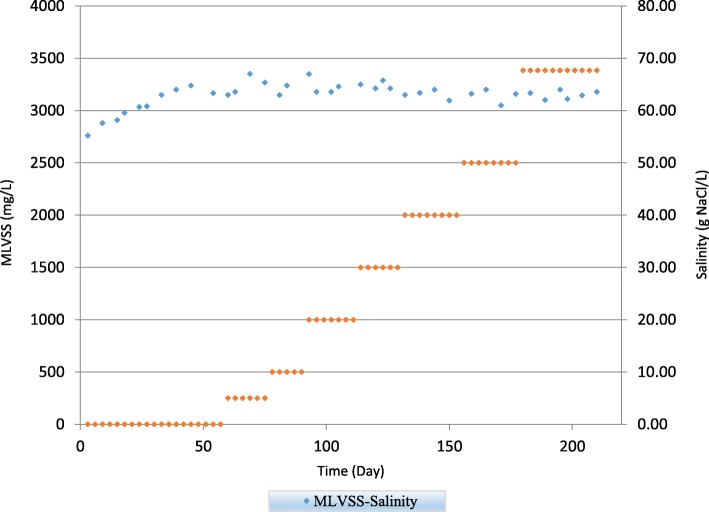
The variation of MLVSS values during whole experiment

The variation of MLVSS values are showed in Fig. 4. It was seen that MLVSS was increased to 3240 mg/L at the second stage of salinity corresponding to 5 g NaCl/L, and then had a relatively constant values until the end of experiments at 67.7 g NaCl/L salinity.

This is suggested that regardless of total mass of microbes whether live cells or dead cells, viable and active cells are remained constant and, accordingly, fixed solid accumulated as salinity rose. As mentioned before, some specific salt-resistant microorganisms which were not dominant species at first, could grow and survived in high saline environment and therefore the amount of viable biomass remained unchanged.

This result was different from the results of Yusoff et al. (2016) [16] where he showed that the MLVSS increases about 80% with increase of salinity in hybrid SBR system. Moussa et al. (2006) [2] reached to the same results compared with present study. He found that the average MLVSS were independent of salt concentrations and remained unchanged at different salt concentrations. Also, Dincer and Kargi (2001) [29] stated that high concentration of salt was led to reduction of specific activity, but not to changes in biomass content. This might result from the diversity of biological treatment process, variety of microorganism in biomass and influent wastewater. Lots of studies have demonstrated that by increasing salt concentration, microbial biodiversity of activated sludge will be greatly reduced (Bond et al., 1995 [30]; Snaidr et al., 1997 [31]; Lefebvre et al., 2006 [32]; Wang et al., 2008 [33]) With increase of the salinity, there were some salt-resistant microorganisms dominated at different salinity levels in activated sludge. Therefore, in high saline environment, all kinds of bacteria in sludge have the trend of reduction. However, with gradual adaptation to salinity, salt-tolerant microbial species will survive and gradually become prevailing microorganisms, and then play a key role in the degradation of saline and hypersaline wastewater (He et al., 2016) [5].

### Variation in sludge settling characteristics

**Fig. 5:**
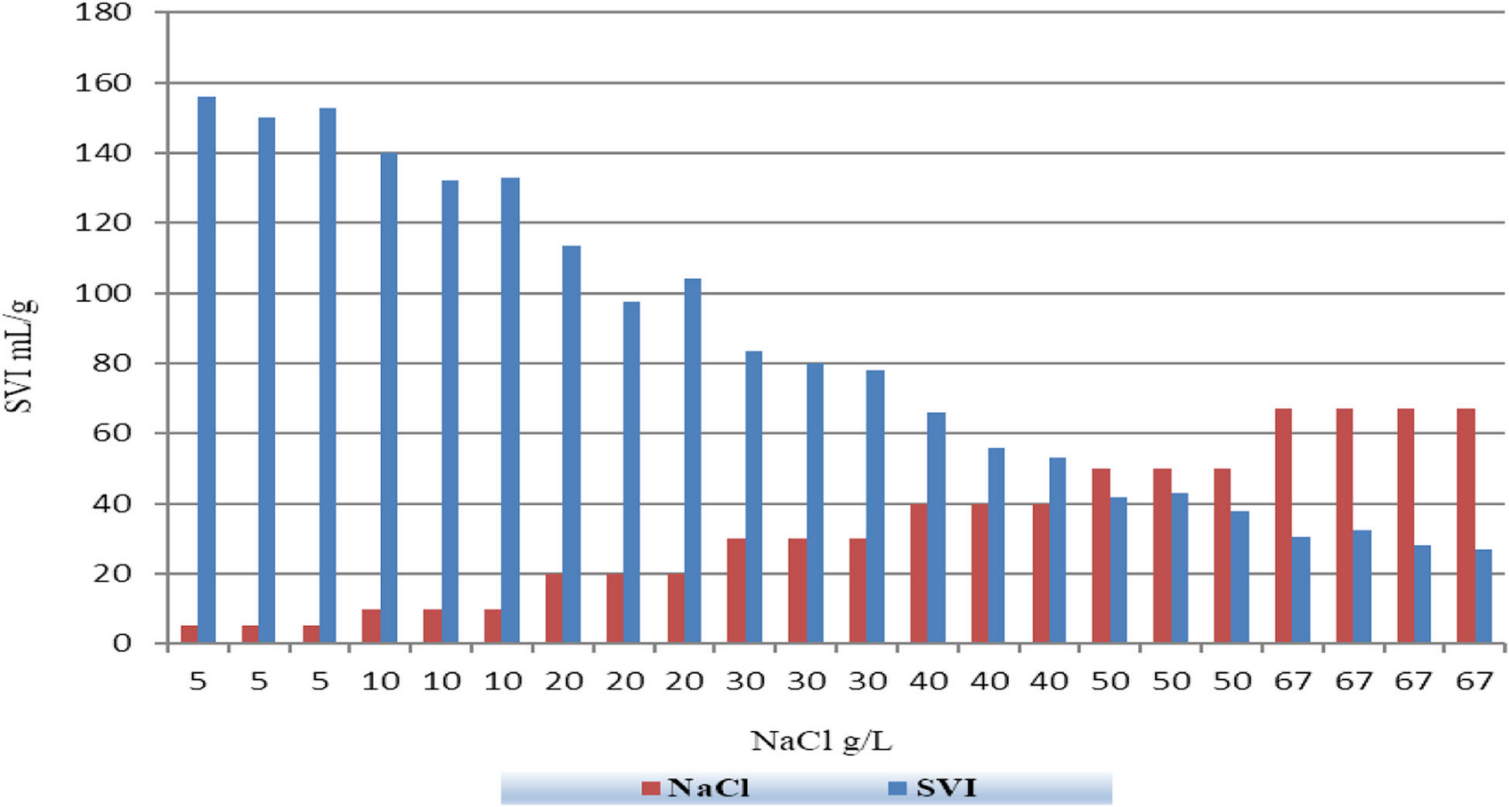
Variation of SVI values in different salinity

The variation of SVI values are represented in Fig. 5. In this study, sludge settling performance was improved by increasing salinity. By increasing salinity from 0 to 67 g NaCl/L the SVI decreased from 156 mL/g to 27 mL/g and this result showed the improvement of sludge settling property with increase of salinity.

**Fig. 6:**
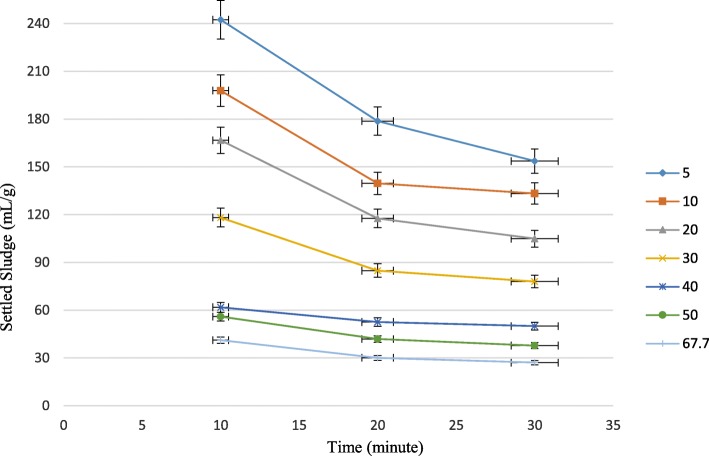
Variation of settled sludge in SVI for different salinities

In addition, sludge settling velocity was increased as shown in Fig. 6. By increasing salinity from 5 to 67.7 g NaCl/L, the differences in settled sludge at 10, 20 and 30-min of SVI experiment became less and less, meaning that the suspended sludge was settled faster. Data represented in Fig. 6 are the average of 3 tests.

The impact of salinity on settling property in activated sludge systems has been subjected to controversial debate over the past few years. In present study, as stated earlier, salinity had a positive effect on settling performance of suspended sludge. However, in some studies, different results were obtained. (Amin et al., 2014 [26]; She et al., 2016 [18]; Uygur and Kargi 2004 [20]; Wang et al., 2016) [15]. They attributed the damaged settling performance of activated sludge with some factor such as, a) reduction of microbial biomass and biodiversity in high saline environment, b) increase of density in wastewater as a result of saline condition and consequently increase of buoyance of suspended solids. C) increasing dispersion of sludge system and so, loss of activated sludge settling property. However, some other studies observed the same results as present study (Moon et al., 2003 [34]; Pronk et al., 2014 [35]; Moussa at al., [2] 2006; Bassin et al., 2012 [36]; Campos et al., 2002 [37] Zhang et al., 2010). Some factor such as a) inhabitation of filamentous bacteria in saline condition which led to better settling conditions b) selection of denser sludge which caused by combination of electrostatic and hydrophobic interactions and consequently reduction of repulsive force between particles c) with salinity activated sludge flocs become smaller and closer d) washout of lighter sludge flocs.

## Conclusion

In present study the capabilities of hybrid growth of bacteria for biological treatment of saline and hypersaline wastewater was investigated. For this purpose, Hybrid SBR with polypropylene suspended carrier, for attached growth was chosen for its simple and compact structure. Results showed, when salinity rose from 0 to 67.7 g NaCl/L, the removal efficiency of COD was constantly increased to 94.22% at 5 g NaCl/L, and then reduced to 91, 87.01, 82.36, 76.27, 65.22% and 52.46 proportional to 10, 20, 30, 40, 50, 67.7 g NaCl/L, respectively. Moreover, MLSS and MLVSS of suspended sludge were measured to evaluate the variation of total and viable suspended solids. It was seen that MLSS increased progressively by 69% during 7 months, however MLVSS was experienced steady improvement until 5 g NaCl/L and then stayed static to the end of experiment. SVI and settling velocity were also measured to assess the settling performance of the system. SVI was decreased from 156 to 27 mL/g and settling velocity showed faster settlement during the whole experiment.

## Data Availability

Please contact author for data requests.

## Abbreviation

COD: Chemical Oxygen Demand
DO: Dissolved Oxygen
EC: Electric Conductivity
EPS: Extra Polymeric Substance
HSBR: Hybrid Sequencing Batch Reactor
MLSS: Mixed Liquor Suspended Solids
MLVSS: Mixed Liquor Volatile Suspended Solids
SBR: Sequencing Batch Reactor
SBU: Shahid Beheshti University
SVI: Sludge Volume Index
